# Mitochondrial calcium uniporter Mcu controls excitotoxicity and is transcriptionally repressed by neuroprotective nuclear calcium signals

**DOI:** 10.1038/ncomms3034

**Published:** 2013-06-18

**Authors:** Jing Qiu, Yan-Wei Tan, Anna M. Hagenston, Marc-Andre Martel, Niclas Kneisel, Paul A. Skehel, David J. A. Wyllie, Hilmar Bading, Giles E. Hardingham

**Affiliations:** 1 Centre for Integrative Physiology, University of Edinburgh, Edinburgh EH8 9XD, UK; 2 Department of Neurobiology, Interdisciplinary Center for Neurosciences (IZN), Im Neuenheimer Feld 364, D-69120 Heidelberg, Germany; 3 These authors contributed equally to this work

## Abstract

The recent identification of the mitochondrial Ca^2+^ uniporter gene (Mcu/Ccdc109a) has enabled us to address its role, and that of mitochondrial Ca^2+^ uptake, in neuronal excitotoxicity. Here we show that exogenously expressed Mcu is mitochondrially localized and increases mitochondrial Ca^2+^ levels following NMDA receptor activation, leading to increased mitochondrial membrane depolarization and excitotoxic cell death. Knockdown of endogenous Mcu expression reduces NMDA-induced increases in mitochondrial Ca^2+^, resulting in lower levels of mitochondrial depolarization and resistance to excitotoxicity. Mcu is subject to dynamic regulation as part of an activity-dependent adaptive mechanism that limits mitochondrial Ca^2+^ overload when cytoplasmic Ca^2+^ levels are high. Specifically, synaptic activity transcriptionally represses *Mcu,* via a mechanism involving the nuclear Ca^2+^ and CaM kinase-mediated induction of Npas4, resulting in the inhibition of NMDA receptor-induced mitochondrial Ca^2+^ uptake and preventing excitotoxic death. This establishes Mcu and the pathways regulating its expression as important determinants of excitotoxicity, which may represent therapeutic targets for excitotoxic disorders.

## Abstract (web summary)

Calcium uptake by the mitochondrial calcium uniporter is implicated in excitotoxicity. This study shows that the uniporter gene product mediates mitochondrial calcium uptake and depolarisation in neurons during excitotoxicity, and is transcriptionally repressed by neuroprotective nuclear calcium signals.

## Graphical abstract

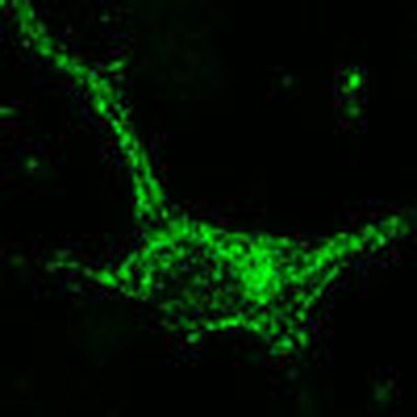

For ~50 years, it has been known that mitochondria are able to take up Ca^2+^, achieved through the action of a membrane potential-driven carrier named the mitochondrial calcium uniporter (Mcu)^1^,^2^. The neurotoxic potential of the excitatory neurotransmitter glutamate has been appreciated for a similarly long time^3^. Glutamate excitotoxicity was found to be due to excessive Ca^2+^ influx through the NMDA subtype of glutamate receptor, and is implicated in promoting neuronal death and dysfunction in a variety of acute and chronic neurological disorders including stroke, traumatic brain injury and Huntington's disease^3^,^4^,^5^,^6^.

Many important studies into the responses of mitochondria to NMDA receptor (NMDAR) activity suggest that mitochondrial Ca^2+^ uptake by the uniporter has a role in excitotoxicity^7^,^8^,^9^. Inappropriate levels of mitochondrial Ca^2+^ uptake, in concert with nitric oxide production and activation of poly(ADP-ribose) polymerase-1 (PARP-1), lead to loss of mitochondrial membrane potential, which in turn energetically compromises the neuron and may lead to ROS generation^7^,^8^,^9^,^10^,^11^. However, a definitive answer to the question of whether mitochondrial Ca^2+^ uptake mediates excitotoxicity has been lacking because the molecular identity of the Mcu was not known. Early attempts to interfere with mitochondrial Ca^2+^ uptake in neurons indirectly involved the use of protonophores in order to depolarize the mitochondria (the membrane potential is essential for uniporter activity)^12^. However, this intervention can dramatically impact the cell’s bioenergetics as well as potentially triggering changes to the plasma membrane potential^7^. Moreover, the protective effects of prior mitochondrial depolarization are controversial^13^. The use of a cocktail of mitochondrial toxins to dissipate the mitochondrial membrane potential, while preventing ATP depletion, has also been employed to indirectly prevent mitochondrial Ca^2+^ uptake, with protective consequences^14^.

Pharmacological agents based on the hexavalent cation ruthenium red have also been utilized. Ruthenium red itself is able to selectively block the uniporter in isolated mitochondria, but has non-selective effects on certain ion channels in intact cells and is unable to cross the plasma membrane of many cell types^15^,^16^. The derivative Ru360 has been proposed to be more selective and cell-permeant (although there remain some doubts in these areas^15^,^16^,^17^). Effects of Ru360 on glutamate-induced mitochondrial depolarization have been observed^11^, although investigations have focussed on early events, as it is unstable in aqueous solutions (it quickly becomes oxidized). Ru360 is of limited use for long-term experiments needed to assess the role of mitochondrial Ca^2+^ uptake in excitotoxic cell death.

In two recent papers, the gene product encoding the uniporter channel (*Mcu*) was identified as the ubiquitously expressed gene previously known as *Ccdc109a*^18^,^19^, which acts in concert with regulatory proteins such as Micu1 and Mcur1 to mediate potential-driven mitochondrial Ca^2+^ uptake^20^,^21^. This finding now allows selective approaches involving exogenous *Mcu* expression and knockdown to be employed to determine the role of mitochondrial Ca^2+^ uptake in all aspects of cellular physiology and pathology.

Here we have manipulated Mcu expression in order to directly investigate the long-standing issue of a role for mitochondrial Ca^2+^ uptake in excitotoxicity. Overexpression and knockdown of Mcu reveals that it has an important role in mitochondrial Ca^2+^ uptake following NMDAR activation, as well as in subsequent cell death. Furthermore, we find that the Mcu gene is subject to dynamic regulation: it is transcriptionally repressed by neuroprotective nuclear Ca^2+^ signals *via* a mechanism involving induction of the transcriptional regulator Npas4.

## Results

### Mcu expression promotes neuronal mitochondrial Ca^2+^ uptake

**Figure 1:**
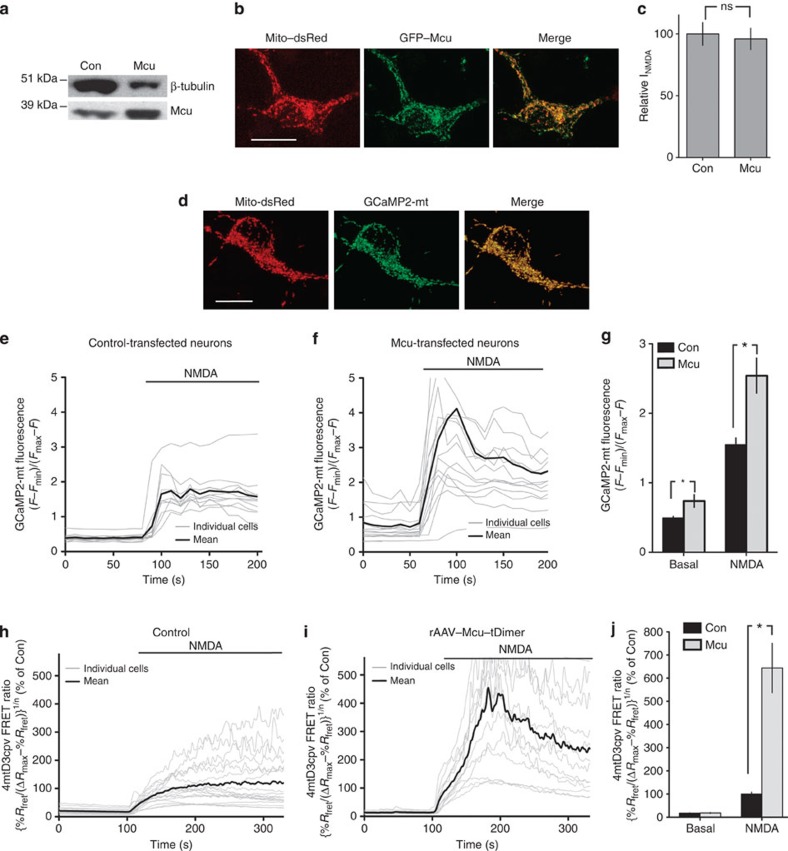
Overexpression of Mcu promotes uptake of Ca^2+^ into mitochondria following NMDA receptor activation. (**a**) Western blot of extracts from control neurons or neurons nucleofected with an Mcu-encoding plasmid. (**b**) Confocal image of a Mito-dsRed and GFP-Mcu co-expressing neuron. Scale bar=15 μm. (**c**) NMDA (150 μM)-evoked whole-cell currents measured in control- and Mcu-expressing neurons (co-expressing GFP for identification) (mean±s.e.m., *n*=7). (**d**) Confirmation that GCaMP2-mt is localized to mitochondria. Scale bar=15 μm. (**e**–**g**) Mcu overexpression boosts mitochondrial Ca^2+^ uptake assayed using GCaMP2-mt. Neurons were transfected with vectors encoding GCaMP2-mt plus either Mcu or control (ß-globin). GCaMP2-mt fluorescence was measured before and during exposure to NMDA (20 μM). (**e**,**f**) show example traces from single experiments involving control (**e**) or Mcu (**f**) over-expressing neurons. For each experiment, the mean value of all cells within the field was calculated (line in bold shown) and the average pre- and post- stimulation level calculated (normalized to the maximal ionomycin-induced signal). 1G shows quantitation of experiments (mean±s.e.m.). *P<0.05 (unpaired two-tailed *t-*test, Con: 45 cells, *n*=6; Mcu: 48 cells, *n*=6). (**h**–**j**) rAAV-mediated expression of Mcu–tDimer boosts mitochondrial Ca^2+^ uptake assayed using 4mtD3cpv. Hippocampal neurons were infected with rAAVs containing vectors encoding 4mtD3cpv±rAAV–*Mcu–tDimer*. 4mtD3cpv FRET ratios were measured before and during NMDA exposure (10 μM) 200–250 s following NMDA application. 1H and 1I show example traces from single experiments involving control (**h**) or rAAV–Mcu–tDimer (**i**) infected neurons. (**j**) shows quantitation (mean±s.e.m.). In all cases, [Ca^2+^] levels obtained using 4mtD3cpv were normalized to the mean NMDA-induced [Ca^2+^] in control-transfected neurons measured on that precise day of imaging. *P<0.05 (unpaired two-tailed *t-*test. Con: 204 cells from *n*=15 experiments; Mcu–tDimer: 100 cells from *n*=12 experiments).

*Mcu* is a ubiquitously expressed gene^19^ (although absent in yeast^2^) and we confirmed expression of Mcu in mouse cortical and hippocampal neurons: western analysis of whole-cell lysates using a previously validated anti-Mcu antibody^18^ revealed a band of expected size that was enriched in neurons over-expressing Mcu (Fig. 1a, Supplementary Fig. S1a). We employed immunofluorescence and biochemical fractionation approaches to show that Mcu fused to the fluorescent proteins eGFP or tDimer localized to neuronal mitochondria, consistent with its known subcellular distribution (Fig. 1b, Supplementary Fig. S1b and data not shown). Our overarching aim was to investigate the effect of manipulating Mcu expression on responses of forebrain neurons to NMDA treatment, focusing on mitochondrial and cytoplasmic Ca^2+^ increases, mitochondrial depolarization, and cell death.

Throughout the study, we present data using two complementary approaches: (a) Liposome-mediated transfection of an Mcu expression vector or Mcu-directed siRNA in mouse cortical neurons, and (b) Recombinant adeno-associated virus (rAAV)-mediated transduction of expression vectors encoding Mcu (tagged with GFP or tDimer) or Mcu-directed shRNA in mouse hippocampal neurons. The reason for the liposome-mediated transfection approach was to achieve low efficiency, enabling a direct comparison between transfected and juxtaposed untransfected cells, while the viral transduction technique was chosen for high efficiency of infection (80–90%), which facilitates ease of data analysis, particularly cell death. The two neuronal types used served to broaden the applicability of our findings.

We first studied the effect of Mcu overexpression on NMDAR-dependent increases in mitochondrial Ca^2+^, loss of mitochondrial membrane potential and cell death. In all Ca^2+^ imaging experiments we performed experiments with control and 'treated' conditions using sister cultures interleaved on the same day to control for any culture-to-culture variation in NMDA-induced Ca^2+^ influx. Thus, the only 'control' data shown in any particular figure are those data collected on the same day on the same culture as when the ‘treated condition’ data (for example, Mcu overexpression) were collected. As an important additional control, we verified that Mcu overexpression did not have any effect on NMDA-evoked whole-cell currents (Fig. 1c).

Rhodamine-based Ca^2+^ indicators are a useful tool for measuring mitochondrial Ca^2+^^22^,^23^, as they partition preferentially into polarized mitochondria due to their positive charge, and responses of transfected and juxtaposed untransfected cells can be analyzed. Neurons loaded with Rhod-2 showed a punctate distribution of the indicator that closely resembled that obtained with mitotracker red^23^ and the pattern was abolished by the mitochondrial uncoupler FCCP (Supplementary Fig. S1c), confirming a predominant mitochondrial localization of the dye. Using Rhod-2 imaging, we found that Mcu over-expressing neurons (co-transfected with an eGFP marker for identification) exhibited a larger NMDA-induced increase in [Ca^2+^]_mit_, compared with surrounding untransfected neurons (Supplementary Fig. S1d,e). In control experiments, neurons transfected with control plasmid (ß-globin) responded no differently than surrounding untransfected neurons (Supplementary Fig. S1d,e).

Although Rhod-2 imaging is well-suited for comparing the responses of adjacent transfected versus untransfected cells, its localization to mitochondria is not completely clear-cut: some cytoplasmic localization is unavoidable, potentially leading to an underestimation of the effects on mitochondrial Ca^2+^. We therefore complemented our Rhod-2 imaging studies with those involving a genetically encoded, mitochondrially targeted Ca^2+^ indicator, GCaMP2-mt^24^ (Fig. 1d). The GCaMP2-mt signal obtained in resting control neurons was ~30% of the maximum signal obtained by ionomycin treatment and the indicator was found to have a *F*_min_ (obtained with ionomycin in zero Ca^2+^ medium) that was barely detectable over background (data not shown). Using this technique, we also found that Mcu overexpression strongly increased both basal [Ca^2+^]_mit_ and NMDA-induced increases in [Ca^2+^]_mit_, compared with control-transfected neurons treated in parallel experiments (Fig. 1e–g).

We next studied the effect of Mcu overexpression on NMDA-induced mitochondrial Ca^2+^ uptake using an rAAV-based approach. We co-infected neurons with an rAAV containing an expression cassette for Mcu (tagged with tDimer for identification) and an rAAV containing an expression cassette for the FRET-based mitochondrial Ca^2+^ indicator 4mtD3cpv^25^. The effect of Mcu overexpression was compared with parallel experiments in which the neurons were infected with only rAAV–*4mtD3cpv*. The rAAV-mediated infection resulted in high efficiency (80–90%) expression of Mcu–tDimer in mouse hippocampal neurons (data not shown). Compared with parallel experiments performed on control neurons Mcu–tDimer overexpression led to enhanced elevation of [Ca^2+^]_mit_, following NMDAR activation (Fig. 1h–j). We have thus shown using independent methods of gene transfer and imaging, that Mcu overexpression in neurons leads to enhanced mitochondrial Ca^2+^ uptake following activation of NMDARs.

### Mcu promotes NMDAR-dependent mitochondrial depolarization

**Figure 2:**
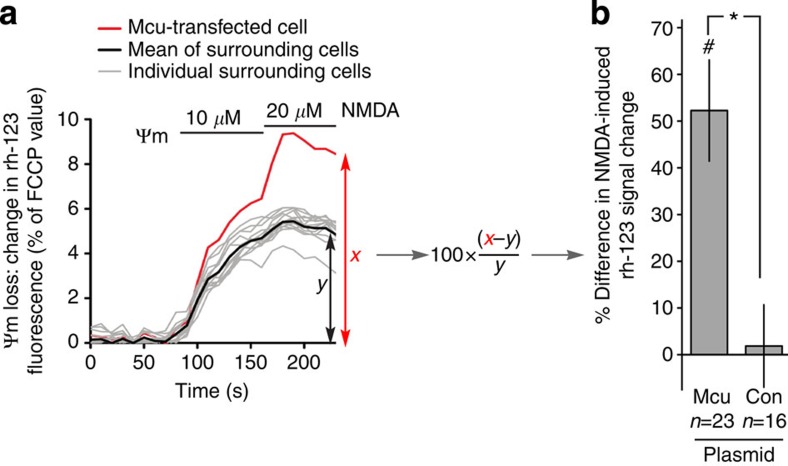
Overexpression of Mcu exacerbates NMDA-induced loss of mitochondrial membrane potential. (**a**,**b**) Example trace from a single experiment where Rhodamine-123 fluorescence changes following NMDA application were studied in a cell overexpressing Mcu (plus an mCherry marker) and surrounding untransfected cells. Rhodamine-123 fluorescence was normalized in all cases to the maximal signal obtained upon complete mitochondrial depolarization, achieved at the end of the experiment by exposure of neurons to the uncoupler FCCP. For each experiment, the difference between transfected and untransfected cells was calculated as shown in the figure, and combined with other repeats to give the quantitation shown in (**b**) (mean±s.e.m.). ^#^ indicates a significant difference between Mcu-expressing cells and surrounding untransfected cells (*P*<0.05, paired two-tailed *t-*test). * indicates a significant difference between data obtained with Mcu-expressing cells and that with control-transfected cells (*P*<0.05, unpaired two-tailed *t-*test, *n*=23 Mcu, *n*=16 Con).

A reduction of mitochondrial membrane potential (Ψm) is one of the earliest observable events in response to excitotoxic insults, and mitochondrial Ca^2+^ uptake is thought to be a key factor in this process^11^,^26^,^27^. We therefore studied the effect of manipulating Mcu expression on NMDA-induced Ψm loss, assayed using the indicator rhodamine-123^10^,^28^. We found that Mcu overexpressing neurons (co-transfected with an expression vector for mCherry for identification of the manipulated cells) exhibited a greater percentage change in rhodamine-123 signal following NMDA treatment, compared with surrounding untransfected neurons, indicative of a greater loss of Ψm (Fig. 2b, see 2a for an example experiment). In control experiments, neurons transfected with control plasmid (expression vector for ß-globin) responded no differently than surrounding untransfected neurons (Fig. 2b). Thus, overexpression of Mcu exacerbates NMDA-induced loss of Ψm, consistent with its effect on mitochondrial Ca^2+^ uptake.

### Mcu enhances vulnerability to NMDAR-dependent excitotoxicity

**Figure 3:**
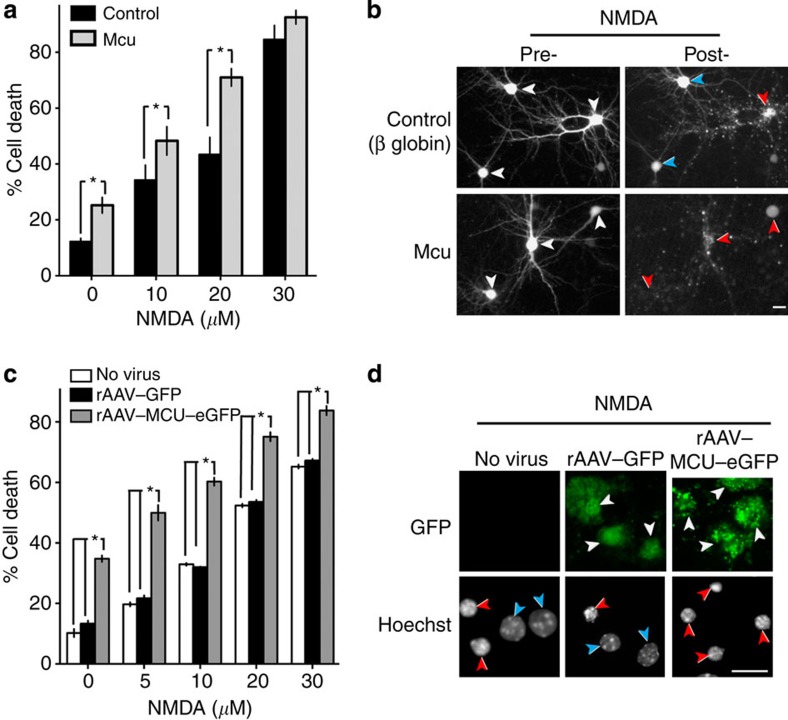
Overexpression of Mcu exacerbates excitotoxic cell death. (**a**,**b**) Cortical neurons were transiently transfected with an eGFP marker plus either control or Mcu-encoding plasmids. Twenty-four hours post-transfection, pictures of transfected cells were taken prior to a 1-h exposure to NMDA (where indicated). After a further 24 h, pictures were taken and cell death scored (mean±s.e.m. shown). *P<0.05 (ANOVA, Bonferroni *post hoc*, 300–500 cells analyzed from *n*=6 independent experiments). Panel (**b**) shows example pictures of cells from (**a**) transfected with the relevant plasmid (+eGFP) pre- and post-NMDA treatment (20 μM). White arrows indicate transfected neurons before NMDA treatment. Red/blue arrows in the ‘post-treatment’ panels indicate dead/live cells, respectively. Scale bar=15 μm (**c**) Hippocampal neurons were infected with the indicated rAAV at DIV 4, and exposed to the indicated concentrations of NMDA at DIV 10, with cell death assessed after 20 h (mean±s.e.m. shown). *P<0.05 (ANOVA, Bonferroni *post hoc*, *n*=3). (**d**) Example pictures from (**c**). Scale bar=15 μm. White arrows indicate infected neurons. Red/blue arrows in the Hoechst panels indicate dead/live cells, respectively. In the rAAV-infected panels, only the infected (eGFP-expressing) neurons are scored as live/dead; in the non-infected control panel, all neurons are scored.

We next investigated the effect of manipulating Mcu expression on the vulnerability of neurons to excitotoxic insults. Pictures of cortical neurons transfected (Lipofectamine) with control or Mcu-encoding vectors plus an eGFP marker were taken before and 24 h after a 1-h exposure to an excitotoxic dose of NMDA, and their viability scored. We found that Mcu overexpression increased the level of basal toxicity in neurons (Fig. 3a). In addition, levels of neuronal death were higher in NMDA-treated neurons over-expressing Mcu compared with control-transfected neurons (Fig. 3a). Similar results were obtained using hippocampal neurons infected with rAAV–*Mcu–GFP*: the expression of Mcu–GFP led to increased basal cell death rates in neurons and elevated levels of NMDA-induced cell death (Fig. 3c). For both cortical and hippocampal neurons, we additionally analyzed the above data to calculate whether Mcu overexpression made neurons more vulnerable to NMDA treatment, taking into account the increased basal death (see Methods). This revealed that Mcu makes neurons significantly more vulnerable to 20 and 30 μM NMDA in cortical neurons and more vulnerable to all doses of NMDA in hippocampal neurons (Supplementary Fig. S2a,b).

### Mcu knockdown impairs Ca^2+^_mit_ and is neuroprotective

**Figure 4:**
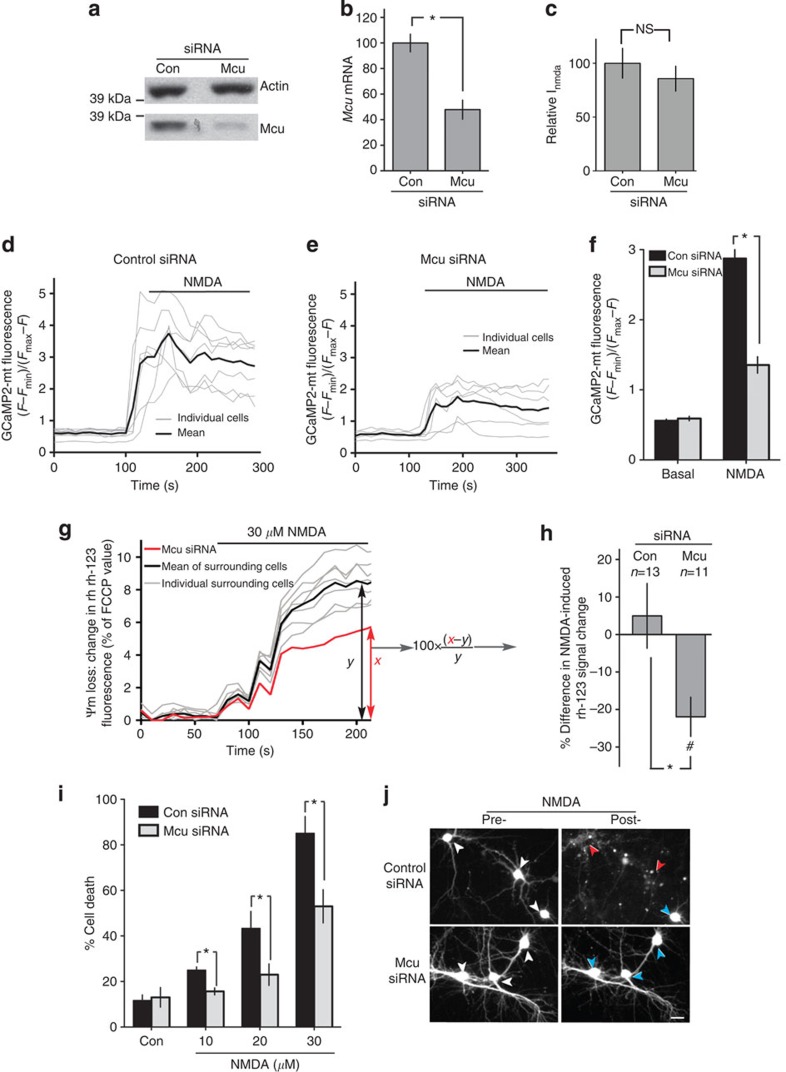
siRNA-mediated knockdown of Mcu suppresses NMDA-induced increases in mitochondrial Ca^2+^ uptake and protects neurons against excitotoxic cell death. (**a**,**b**) Efficacy of Mcu-directed siRNA assessed by nucleofecting Mcu or control siRNA into cortical neurons and assessing Mcu protein (**a**) or mRNA (**b**) expression (*n*=4). *P<0.05 (paired two-tailed *t-*test). Mean±s.e.m. shown. (**c**) NMDA-evoked whole-cell currents measured in neurons transfected with the indicated siRNAs (co-expressing eGFP for identification, *n*=5). Mean±s.e.m. shown. (**d**–**f**) Mcu knockdown boosts mitochondrial Ca^2+^ uptake assayed using GCaMP2-mt. Methodology used as per Fig. 1e–g except that NMDA was used at 30 μM. *P<0.05 (paired two-tailed *t-*test, Con siRNA: 33 cells analyzed from *n*=5 experiments; Mcu siRNA: 31 cells analyzed from *n*=5 experiments). Mean±s.e.m. shown. (**g**,**h**) Mcu knockdown reduces NMDA-induced loss of mitochondrial membrane potential. Mean±s.e.m. shown. Methodology used as in Figure 2a, except that 30 μM NMDA was used. ^#^*P*<0.05 (unpaired two-tailed *t-*test) indicates a significant difference between Mcu siRNA-transfected cells and surrounding untransfected cells. *P<0.05 (unpaired two-tailed *t-*test) indicates a significant difference between data obtained with Mcu siRNA-transfected cells and that obtained with control siRNA-transfected cells (*n*=13 Con siRNA, *n*=11 Mcu siRNA). (**i**,**j**) SiRNA-mediated Mcu knockdown protects neurons against excitotoxic cell death. Methodology used as per Fig. 3a. *P<0.05 (paired two-tailed *t-*test, 200–400 cells analyzed from *n*=4 independent experiments). Mean±s.e.m. shown. Panel (**j**) shows example pictures of cells from (**i**) transfected with the relevant siRNA (+eGFP) pre- and post-NMDA treatment (30 μM). White arrows indicate transfected neurons before NMDA treatment. Red/blue arrows in the ‘post-treatment’ panels indicate dead/live cells, respectively. Scale bar=15μm.

**Figure 5:**
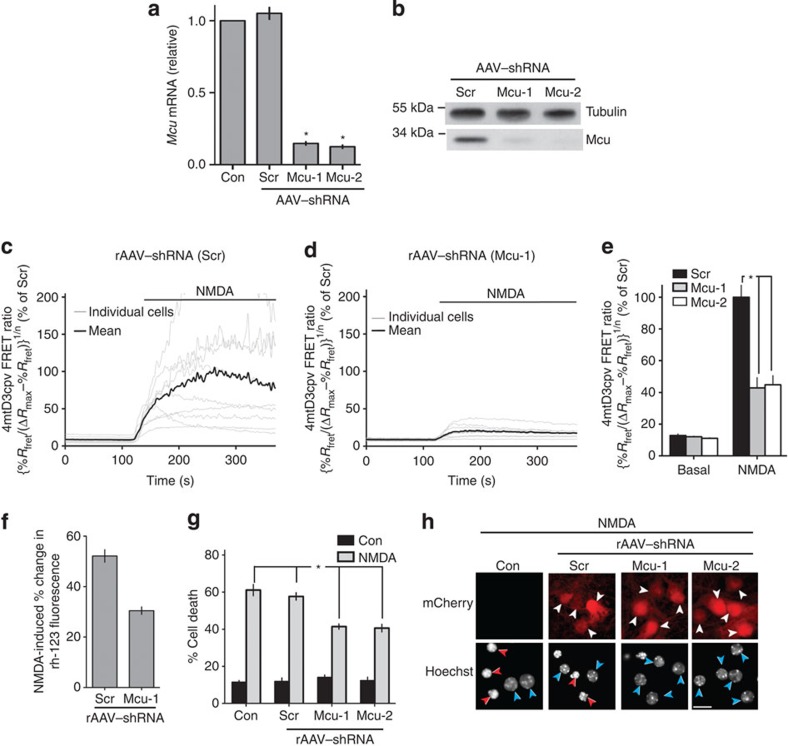
ShRNA-mediated knockdown of Mcu suppresses NMDA-induced increases in mitochondrial Ca^2+^ uptake and protects neurons against excitotoxic cell death. (**a**,**b**) Efficacy of Mcu-directed shRNA assessed by qRT PCR for mRNA (**a**) or western blot for protein (**b**) expression. Hippocampal neurons were infected with rAAVs containing a vector encoding shRNA directed against Mcu (two sequences: Mcu-1 and Mcu-2) or scrambled sequence shRNA (Scr). Knockdown was assessed 6 days post-infection. *P<0.05 (ANOVA, Bonferroni *posthoc*, *n*=3). Mean±s.e.m. shown. (**c**–**e**) rAAV-mediated expression of Mcu-directed shRNAs suppresses mitochondrial Ca^2+^ uptake assayed using 4mtD3cpv. Hippocampal neurons were infected with rAAVs containing a vector encoding 4mtD3cpv, plus rAAV–shRNA encoding Mcu-directed shRNA or scrambled sequence shRNA (Scr). 4mtD3cpv FRET ratios were measured before and during exposure to NMDA (10 μM) 200–250 seconds following NMDA application. Panels (**c**) and (**d**) show example traces from single experiments involving neurons expressing Scr (**c**) or Mcu (**d**)-directed shRNA. Panel (**e**) shows quantitation of all experiments performed (mean±s.e.m.). In all cases, [Ca^2+^] levels obtained using 4mtD3cpv were normalized to the mean NMDA-induced [Ca^2+^] in Scr-transfected neurons measured on that precise day of imaging. *P<0.05 (ANOVA, Bonferroni *post hoc*. Scr: 68 cells from *n*=9 experiments; Mcu-1: 130 cells from *n*=10 experiments; Mcu-2: 85 cells from *n*=10 experiments). (**f**) Hippocampal neurons were infected with the indicated rAAV at DIV4, and at DIV 10 subjected to Rhodamine-123 based mitochondrial membrane potential imaging. Percent changes in Rhodamine-123 fluorescence upon NMDA (30 μM) application were measured. *P<0.05 (Scr: 211 cells from 13 experiments; Mcu-1: 257 cells from 13 experiments). (**g**) Hippocampal neurons were infected with the indicated rAAV at DIV4, and exposed to the indicated concentrations of NMDA at DIV 10, with cell death assessed at 24 h. *P<0.05 (paired two-tailed *t-*test, *n*=4). Mean±s.e.m. shown. (**h**) Example pictures from (**g**). White arrows indicate infected neurons. Red/blue arrows in the Hoechst panels indicate dead/live cells, respectively. In the rAAV-infected panels, only the infected (mCherry-expressing) neurons are scored as live/dead; in the non-infected control panel, all neurons are scored.

We then performed loss-of-function experiments to investigate the effect of interfering with endogenous Mcu expression on NMDAR-dependent increases in mitochondrial Ca^2+^, loss of mitochondrial membrane potential and cell death. Two independent approaches were taken: liposome-mediated transfection of *Mcu*-targeted pre-made siRNA, and rAAV-mediated expression of *Mcu*-directed small hairpin RNA (shRNA). The efficacy of the pre-made siRNA was investigated using a high-efficiency transfection approach (nucleofection), which revealed a partial (50%) knockdown at protein and mRNA levels (Fig. 4a, Supplementary Fig. S1a) and no effect on NMDA-evoked whole-cell currents (Fig. 4c). Given the nucleofection efficiency is never greater than 70–80%, the 50% knockdown reflects a >50% knockdown in transfected cells. We found that neurons transfected with Mcu-directed siRNA exhibited a smaller NMDA-induced increase in [Ca^2+^]_mit_, assayed either using the Rhod-2 indicator and comparing the results to surrounding untransfected neurons (Supplementary Fig. S3a,b), or using GCaMP2-mt and comparing the results to control-transfected neurons (Fig. 4d-f). Neurons transfected with Mcu-directed siRNA also exhibited a smaller loss of Ψm than surrounding cells, while control-transfected neurons did not show differences compared with surrounding cells, (Fig. 4g). We then investigated whether Mcu knockdown influenced NMDA-induced excitotoxicity, and found this indeed to be the case: levels of NMDA-induced death were lower than in control-transfected cells (Fig. 4i). Moreover, we confirmed that the mitochondrial membrane potential of surviving cells (24 h after the NMDA insult) was intact and no different from control cells (Supplementary Fig. S3c). We also confirmed that the resting cytoplasmic Ca^2+^ concentration of surviving cells was low and no different from that of control cells (Supplementary Fig. S3d). This showed that protection due to Mcu knockdown is (as expected) associated with the long-term preservation of mitochondrial capacity and Ca^2+^ homeostasis. Additionally, we found that the effect of Mcu-directed siRNA on neuronal survival was abolished by overexpression of a specially engineered siRNA-resistant form of Mcu, confirming the specificity of the effect (Supplementary Fig. S3e,f). Using the rAAV-mediated expression of Mcu-directed shRNAs, we achieved a >90% knockdown of Mcu (Fig. 5a, Supplementary Fig. S1aii), which when compared with control neurons (infected with an rAAV containing an expression cassette for a scrambled control shRNA) led to a reduction in NMDA-induced [Ca^2+^]_mit_ increases (Fig. 5c), a reduction in NMDA-induced mitochondrial membrane depolarization (Fig. 5f), and protection against NMDA-induced excitotoxicity (Fig. 5g).

### Mcu is transcriptionally repressed by synaptic activity

**Figure 6:**
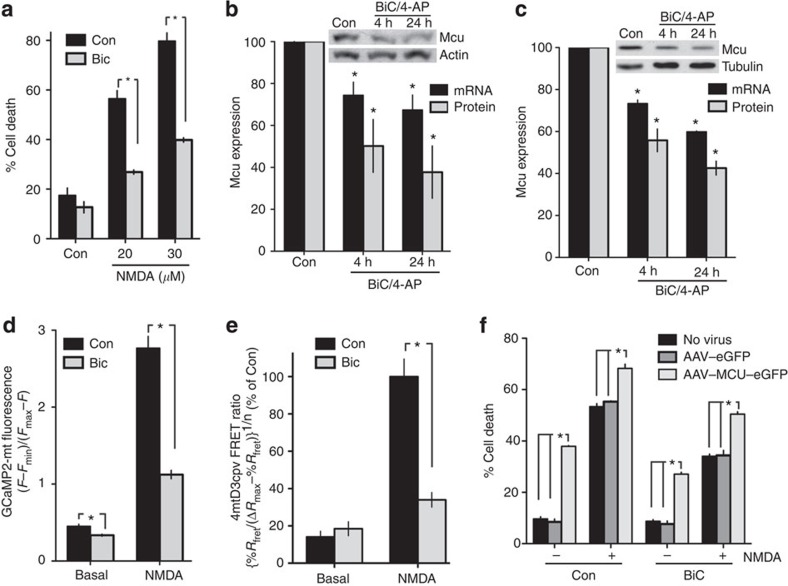
Repression of Mcu-mediated mitochondrial Ca^2+^ uptake by neuroprotective synaptic activity. (**a**) Cortical neurons were pre-treated overnight with bicuculline (50 μM) plus 4-aminopyridine (250 μM) (BiC/4-AP, BiC in graph legends), which was removed and replaced with medium containing the indicated concentrations of NMDA. After 1 h, NMDARs were blocked by the application of MK-801 and cell death levels were assessed 23 h later by fixing and DAPI-staining the cells, and counting nuclear pyknosis. *P<0.05 (ANOVA, Bonferroni *post hoc*, 700–1000 cells analyzed from *n*=3 independent experiments). Mean±s.e.m. shown. (**b**,**c**) Mcu expression following BiC/4-AP treatment analyzed by qRT–PCR (*n*=3) and western blot (*n*=4) in both cortical (**b**) and hippocampal (**c**) neurons. Mean±s.e.m. shown. (**d**) Synaptic activity suppresses NMDA-induced mitochondrial Ca^2+^ uptake. Neurons were transfected with vectors encoding GCaMP2-mt and then treated±BiC/4-AP as in (**a**). Cells were then transferred to fresh medium and GCaMP2-mt fluorescence imaged before and after NMDA treatment (30 μM). For each experiment, the mean value of all cells within the field was calculated and the average pre- and post- stimulation level calculated (normalized to the maximal ionomycin-induced signal). *P<0.05 (unpaired two-tailed *t-*test. Con: 22 cells, *n*=4; BiC/4-AP: 43 cells, *n*=4). Mean±s.e.m. shown. (**e**) Synaptic activity suppresses NMDA-induced mitochondrial Ca^2+^ uptake using 4mtD3cpv. Hippocampal neurons were infected with rAAVs containing a vector encoding 4mtD3cpv and treated±BiC overnight. 4mtD3cpv FRET ratios were measured before and during exposure to NMDA (10 μM) 200–250 s following NMDA application. Shown is the quantitation of all experiments performed. In all cases, [Ca^2+^] levels obtained using 4mtD3cpv were normalized to the mean NMDA-induced [Ca^2+^] in control-transfected neurons measured on that precise day of imaging. *P<0.05 (paired two-tailed *t-*test. Con: 201 cells from *n*=16 experiments; BiC: 224 cells from *n*=16 experiments). Mean±s.e.m. shown. (**f**) Neurons infected with the indicated AAVs were stimulated where indicated with BiC for 16 h followed by exposure to fresh medium±NMDA. Cell death was assessed after a further 20 h. *P<0.05 (paired two-tailed *t-*test, *n*=3). Mean±s.e.m. shown.

It has long been known that episodes of synaptic activity can promote neuroprotection against excitotoxic insults^23^,^29^,^30^,^31^. While multiple parallel mechanisms are likely to mediate this effect, the key role of Mcu-driven mitochondrial Ca^2+^ uptake in excitotoxic cell death led us to investigate whether neuroprotective electrical activity influences Mcu-mediated effects. Burst activity was initiated in cortical neurons by treatment with the GABA_A_ receptor antagonist bicuculline in the presence of the weak K^+^ channel blocker 4-aminopyridine (BiC/4-AP), as employed previously^32^,^33^. Consistent with previous studies, we found that BiC/4-AP pre-treatment, followed by wash-out, rendered neurons resistant to subsequently applied excitotoxic doses of NMDA (Fig. 6a). Strikingly, we also observed that the same stimulation resulted in the downregulation of Mcu expression at both protein and mRNA levels in cortical and hippocampal neurons (Fig. 6b).

This raises the exciting possibility that neuroprotective synaptic activity may actually influence the degree of mitochondrial Ca^2+^ uptake following subsequent NMDA treatment. Indeed, this was found to be the case: mitochondrial Ca^2+^ uptake during NMDA treatment was substantially reduced in BiC/4-AP pre-treated cortical neurons (Fig. 6d) and BiC pre-treated hippocampal neurons (Fig. 6e). Importantly, BiC/4-AP pre-treatment of neurons did not reduce NMDAR whole-cell currents (Supplementary Fig. S4a) and moreover, Ca^2+^ imaging using a non-mitochondrially targeted, cytoplasmic GCaMP2 revealed that basal and NMDA-induced cytoplasmic Ca^2+^ levels were not reduced by BiC/4-AP pre-treatment (Supplementary Fig. S4b). In fact, they were in both cases slightly larger, potentially a consequence of reduced mitochondrial Ca^2+^ uptake. Thus, compared with control, neurons that had recently experienced strong synaptic activity showed a striking difference in the relationship between cytoplasmic Ca^2+^ increases and subsequent mitochondrial Ca^2+^ uptake. Collectively these data support a model whereby prior firing activity suppresses toxic mitochondrial Ca^2+^ uptake following cytoplasmic Ca^2+^ increases, at least in part via the repression of Mcu expression. Of note, we found that forced expression of Mcu promotes increased vulnerability to excitotoxicity even in synaptically active neurons (Fig. 6f). In other words, Mcu overexpression increases the level of neuronal death to a point that is that is beyond the protective effects of synaptic activity.

### Mcu is repressed by nuclear Ca^2+^-dependent CaM kinases

Finally, we investigated the mechanism by which synaptic activity represses Mcu expression. The observed lowering of *Mcu* mRNA in response to synaptic activity was suggestive of either transcriptional repression or reduced mRNA stability (that is, elevated degradation rate). We studied mRNA stability using the standard method of inhibiting transcription with Actinomycin D and harvesting RNA at multiple time points thereafter. By comparing the level of Mcu mRNA with a relatively stable mRNA (18S), one can gain a measure of mRNA degradation, and assess the influence of prior synaptic activity on this stability. An episode of prior synaptic activity had no significant difference in the decay kinetics of Mcu mRNA levels (Supplementary Fig. S5). This result strongly suggests that repression of *Mcu* mRNA levels is due to the repression of transcription.

**Figure 7:**
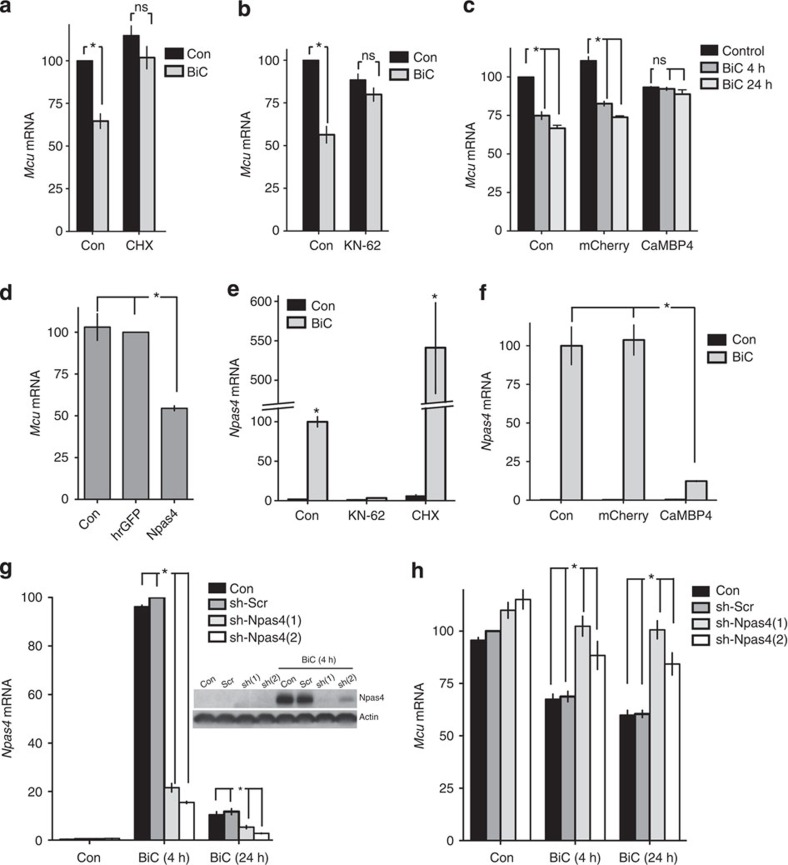
Activity-dependent repression of Mcu requires the nuclear Ca^2+^ and CaM kinase-dependent induction of immediate-early gene *Npas4*. (**a**) qPCR analysis of Mcu expression in neurons treated±BiC/4-AP in the presence or absence of cycloheximide (CHX, 10 μg per ml), applied 1 h prior to BiC/4-AP *P<0.05 (paired two-tailed *t-*test, *n*=3). Mean±s.e.m. shown. (**b**) qPCR analysis of Mcu expression in neurons treated±BiC/4-AP in the presence or absence of a CaM kinase inhibitor (KN-62, 10 μM), applied 1 h prior to BiC/4-AP *P<0.05 (paired two-tailed *t-*test, *n*=3). Mean±s.e.m. shown. (**c**) Neurons were infected where indicated with rAAVs containing a vector encoding mCherry or CaMBP4, followed by BiC/4-AP treatment for the indicated times. *P<0.05 (ANOVA, Bonferroni *post hoc*, *n*=3). Mean±s.e.m. shown. (**d**) Neurons were infected with rAAV containing a vector encoding hrGFP control or Npas4 prior to RNA extraction and qPCR analysis for Mcu levels. *P<0.05 (paired two-tailed *t-*test, *n*=6). (**e**) Neurons were treated with the indicated drugs prior to 4 h BiC/4-AP treatment prior to RNA extraction and qPCR analysis for Npas4 levels. *P<0.05 (paired two-tailed *t-*test, *n*=3). Mean±s.e.m. shown. (**f**) Neurons treated as in (**c**) and Npas4 levels studied (BiC/4-AP treatment for 4 h). *P<0.05 (paired two-tailed *t-*test, *n*=3). Mean±s.e.m. shown. (**g**) Neurons were infected with rAAV-containing vectors encoding the indicated shRNAs targeting Npas4 (or control scrambled shRNA), and the effect of this on BiC/4-AP induction of Npas4 mRNA (graph) or protein (inset) measured, *P<0.05 (ANOVA, Bonferroni *post hoc*, *n*=5). Mean±s.e.m. shown. (**h**) As for 7 g, except that Mcu expression was measured. *P<0.05 (ANOVA, Bonferroni *post hoc*, *n*=5). Mean±s.e.m. shown.

We then investigated the mechanism of this transcriptional repression. We found that activity-dependent repression of Mcu was blocked by cycloheximide, indicative that *de novo* gene expression is required (that is, Mcu regulation is not ‘immediate-early’ in nature, Fig. 7a). Moreover, repression of Mcu by synaptic activity was blocked both by CaM kinase inhibition with KN-62 (Fig. 7b) and also by the nuclear-localized Ca^2+^/Calmodulin inhibitor CaMBP4^33^,^34^,^35^ (Fig. 7c). This suggested a mechanism of Mcu repression that involved the nuclear Ca^2+^ and CaM kinase-dependent induction of a gene product that in turn (directly or indirectly) represses Mcu expression.

### Activity-dependent Mcu repression requires Npas4 induction

Our laboratories have previously described the neuroprotective effects of nuclear Ca^2+^ signaling^33^ and also discovered a series of nuclear Ca^2+^-induced neuroprotective genes, five of which were found to inhibit NMDA-induced mitochondrial depolarization^35^. We therefore screened these genes for an ability to repress Mcu expression and found that viral overexpression of Npas4 was sufficient to repress Mcu expression (Fig. 7d). Npas4 is a transcription factor that can promote negative gene regulation in neurons^36^, and it is an immediate-early gene: its induction by synaptic activity was not reduced by protein synthesis inhibition (cycloheximide treatment, Fig. 7e). Indeed, superinduction in the presence of cycloheximide was observed as is common with immediate-early genes. Moreover, Npas4 induction by synaptic activity was strongly inhibited by both KN-62 and CaMBP4 (Fig. 7e), the same interventions that prevented Mcu repression (Fig. 7b). To directly test whether Npas4 induction was required for activity-dependent Mcu repression, we performed shRNA-mediated knockdown of Npas4 (Fig. 7g) and found that this inhibited activity-dependent repression of Mcu (Fig. 7h). Thus, activity-dependent Mcu repression involves the nuclear Ca^2+^ and CaM kinase-dependent induction of Npas4, which in turn mediates the transcriptional repression of Mcu. These data establish Npas4 as a potentially central regulator of Mcu activity in neurons.

## Discussion

In this study, we used gain- and loss-of-function experiments to demonstrate the central role of the *Mcu* gene product in the control of mitochondrial Ca^2+^ uptake in neurons following an excitotoxic insult. Moreover, we established that Mcu is an important mediator of death signal-induced loss of mitochondrial membrane potential and relays NMDA receptor stimulation to excitotoxic cell loss. Intriguingly, we also find that Mcu expression and mitochondrial Ca^2+^ uptake are subject to dynamic regulation by neuroprotective firing activity.

Several key pro-death pathways are activated in parallel in response to an excitotoxic insult, including nitric oxide and ROS production, oxidative stress, JNK and PARP-1 activation, and CREB shut-off^3^,^37^,^38^,^39^,^40^. Their relative importance may depend on the cell type, developmental stage, and severity of insult. Nevertheless, in most scenarios, it is likely that more than one pathway is important and that pathways can interact with each other. Future studies on Mcu-driven Ca^2+^ uptake will tell us what role this process has in several key pro-death cascades. For example, mitochondrial Ca^2+^ uptake is implicated in the activation of PARP-1 *via* mitochondrial ROS generation, which combine with NO to trigger ONOO^−^-mediated DNA damage, the trigger for PARP-1 activation^11^,^41^,^42^. Moreover, mitochondrial Ca^2+^ uptake and ROS production have also been implicated in JNK activation^43^. Another important issue surrounds the contribution of factors other than mitochondrial Ca^2+^ uptake in mitochondrial membrane depolarization. The coupling of the GluN2B C-terminal domain to downstream cell death pathways, including nitric oxide production, contributes to excitotoxicity^3^,^44^,^45^,^46^, and excitotoxic mitochondrial depolarization via Ca^2+^ uptake has been reported to involve nitric oxide^10^,^47^. Nitric oxide sensitizes mitochondria to Ca^2+^-dependent depolarization^10^, raising the possibility that it affects mitochondrial energy metabolism, Mcu activity itself, or even efflux via the mitochondrial Na^+^/Ca^2+^ exchanger. PARP-1 has also been shown to contribute to excitotoxic mitochondrial depolarization, likely via depletion of NADH levels^11^. Despite the existence of multiple mechanisms leading to excitotoxicity, it is thus apparent that there is crosstalk between pathways and that mitochondrial dysfunction is a convergent hub for some and an upstream effector for others. In seeking to minimize neuronal death and dysfunction in excitotoxic disorders such as stroke, targeting multiple sites of control may be optimal. One can envisage that targeting upstream (big effect, short therapeutic window) as well as downstream (smaller effect, longer window) events could be more effective than any one strategy. Future studies will help to illuminate and clarify these issues.

It will also be of interest to determine whether and how mitochondrial Ca^2+^ uptake contributes to the differential effects of synaptic versus extrasynaptic NMDAR activity^28^,^38^. Extrasynaptic NMDAR activity is particularly well-coupled to mitochondrial depolarization^28^, and Ca^2+^ influx triggered by bath activation of NMDARs is more strongly coupled to mitochondrial Ca^2+^ uptake than influx through other routes^27^. It is tempting to speculate that physical proximity of extrasynaptic NMDARs to mitochondria may contribute to this. Given the relatively low affinity and huge Ca^2+^-carrying capacity of the uniporter^1^ (half-saturation reached at 20 mM Ca^2+^), Ca^2+^ uptake may be substantially enhanced if mitochondria are positioned extremely near the site of Ca^2+^ entry where local Ca^2+^ levels are high. Synaptic NMDARs may be somewhat shielded from close proximity to mitochondria by the post-synaptic density, and more significantly by the fact that dendritic spines rarely contain mitochondria^48^. It will also be important to determine whether other mitochondrial Ca^2+^ transporters such as Letm1 or uncoupling proteins contribute in any way in neurons^49^,^50^, and which Mcu regulatory proteins (for example, Micu1/2 and Mcur1) are important. We occasionally observed biphasic kinetics in the increase of [Ca^2+^]_mit_ (for example, see Fig. 1i), potentially indicating functionally different uptake mechanisms.

Our findings suggest that pharmacological modulation of Mcu offers a realistic therapeutic target for excitotoxic injury, as ischemia-induced excitotoxicity is likely to develop over a relatively long timescale. Interference with early events in the excitotoxic cascade can yield protective outcomes, such as those employing NMDAR antagonists, and PSD-95 and JNK inhibitors^3^,^37^,^43^,^46^,^51^. Now that the molecular machinery of the Mcu has been identified^18^,^19^, and its role in excitotoxicity established (this study), screens for small molecules that inhibit Mcu activity may lead to novel neuroprotective compounds. Of course, the appropriateness of the Mcu as a therapeutic target needs to be assessed in the light of all its physiological role(s). Evidence supports the notion that mitochondrial Ca^2+^ controls mitochondrial energy metabolism, including in neurons^52^,^53^. The impact of interfering with Mcu activity on these processes awaits further investigation, although we note from the current study that Mcu knockdown has no adverse effects on basal viability.

Our observations that Mcu is subject to transcriptional repression by neuroprotective Ca^2+^ signals may reflect an important facet of Mcu’s function in the cell. As Mcu-mediated mitochondrial Ca^2+^ overload contributes to mitochondrial dysfunction and neuronal death in response to excitotoxic insults, repression of Mcu expression by Ca^2+^ signals may represent a potentially important feedback mechanism acting to prevent mitochondrial Ca^2+^ overload, (potentially in concert with other mechanisms^54^). It is tempting to speculate that this mechanism enables a cell to tune its Mcu activity to reflect cytoplasmic Ca^2+^ levels, and that Mcu transcriptional repression is an important factor in Ca^2+^-dependent tolerance/preconditioning mechanisms.

Protection against excitotoxic insults by Ca^2+^-dependent transcriptional changes has centered mainly on the Ca^2+^ responsive transcription factors involved. Induction of CREB-mediated transcription and inhibition of FOXO-mediated gene expression have been shown to contribute to protection against excitotoxic insults by neuroprotective Ca^2+^ signals^29^,^31^. Our observation that repression of Mcu expression underlies an important part of the anti-excitotoxic effects of sub-toxic Ca^2+^ signals demonstrates that a central effector of excitotoxicity is under direct control by neuroprotective pathways. Furthermore, we have demonstrated that this repression is a delayed response to the immediate-early induction of Npas4, although whether Npas4 is acting directly on the Mcu promoter in an inhibitory capacity or acting indirectly (for example, by inducing the expression of a repressor) awaits further investigation. Given that the *MCU* gene is also subject to Ca^2+^-mediated transcriptional repression in human ESC-derived neurons (Qiu and Hardingham, unpublished observations), a search for phylogenetically conserved elements within the Mcu promoter may yield the sequences responsible for mediating Ca^2+^ sensitivity.

To conclude, we have established *Mcu* both as an effector of excitotoxic cell death and a target of neuroprotective signals. Exploiting these facts may lead to new interventions aimed at ameliorating neuronal dysfunction and death in disorders associated with aberrant NMDAR activity.

## Methods

### Neuronal culture

Hippocampal and cortical neurons from newborn C57Bl/6 mice (sex not determined) were cultured and maintained as described previously^33^,^35^. Neurons were cultured in Neurobasal media (Invitrogen, Gaithersburg, MD, USA) containing 1% rat serum and B27 (Invitrogen), and penicillin/streptomycin. Experiments were performed after a culturing period of 10–13 days during which cultured neurons develop a rich network of processes, express functional NMDA-type and AMPA/kainate-type glutamate receptors, and form synaptic contacts.

### Transfection and nucleofection

Neurons were transfected in trophic transfection medium^55^ with plasmids (2 μg ml^−1^ total) and/or siRNA (100 nM) using Lipofectamine 2000. Nucleofection was performed using the Amaxa rat Neuron Nucleofector Kit (Lonza). See Supplementary Methods for further details and siRNA target sequences.

### Western blotting and Subcellular fractionation

Gel electrophoresis and western blotting were performed using the Xcell Surelock system (Invitrogen) using precast gradient gels (4–20%) as described^45^. The following antibodies were used: Ccdc109a (Sigma, 1:500), ß-actin (Abcam, 1:2,000), α-Tubulin (Sigma, 1:400,000), anti PDH-E1-alpha (Abcam, 1:1,500); anti C-V-alpha (Abcam, 1:1,500); anti Erk 1/2 (NEB, 1:5,000). For visualization of western blots, HRP-based secondary antibodies were used followed by chemiluminescent detection on Kodak X-Omat film. Western blots were analyzed by digitally scanning the blots, followed by densitometric analysis (ImageJ). For figure preparation of western blots, linear adjustment of brightness/contrast was applied (Photoshop) equally across the image. See Supplementary Methods for details of subcellular fractionation.

### Plasmids and virus generation

The vector containing the mouse CaMKIIα promoter used to construct and package rAAV has been described previously^56^. For knockdown using shRNA, we used a rAAV vector containing the U6 promoter driving shRNA expression and that also contained a CaMKII promoter driving mCherry expression (to identify infected neurons). See Supplementary Methods for details of cloning and sequences used to generate rAAV vectors encoding Mcu, 4mtD3cpv and shRNA targeting *Mcu* and *Npas4*.

Neurons were infected with 10^11^ rAAV particles per μl at DIV 4. Infection efficiencies were determined at DIV 10 by analyzing the fluorescence of eGFP or mCherry; they ranged from 80 to 90% of the viable neurons. rAAV expression vectors for Npas4, CaMBP4 and mCherry have been described previously^35^,^56^. Mcu-GFP was a gift from Vamsi Mootha^18^, pCAGGS-GCaMP2 was a gift from Karel Svoboda^57^; GCaMP2-mt was a gift from Xianhua Wang^24^, Mt-cameleon-pcDNA3 (containing 4mtD3cpv cDNA) was a gift from Roger Tsien^25^.

### Electrophysiological recording and analysis

NMDA-evoked whole-cell steady-state currents (normalized to cell capacitance) were measured 48 h after transfection as described^45^. NMDA (150 μM) was applied for 30 s, repeated twice for each cell. Data were filtered at 1 kHz and digitized at 5 kHz for subsequent off-line analysis.

### Mitochondrial Ca^2+^ imaging

In all Ca^2+^ imaging experiments, we performed experiments with control and 'treated' conditions using sister cultures interleaved on the same day to control for any culture-to-culture variation in NMDA-induced Ca^2+^ influx. Thus, the only 'control' data shown in any particular figure are those data collected on the same day on the same culture as when the 'treated condition' data (for example, Mcu overexpression) were collected. Several imaging approaches were used: (a) Rhod-2 imaging: this was performed as described^23^ using a Leica AF6000 LX imaging system, with a DFC350 FX digital camera. Neurons loaded with Rhod-2 showed a punctate distribution of the indicator that very closely resembled that obtained with mitotracker red (data not shown) and the pattern was abolished by the mitochondrial uncoupler FCCP (Supplementary Fig. S1c), confirming the mitochondrial localization of the dye. Transfected cells were identified by co-transfecting eGFP expression plasmid. The NMDA-induced increase in Rhod-2 signal (ΔF/F, ex 546±6 nm; em 600±20 nm) of each transfected cell was compared to that observed in surrounding untransfected cells in a 100 μm radius and subsequently expressed as a percentage of that observed in those untransfected cells. (b) GCaMP2-mt imaging: neurons were transfected with GCaMP2-mt-encoding vector, the fluorescence signal of which was detected using a standard GFP filter set (ex 480±20; em 527±15). NMDA-induced changes in mitochondrial Ca^2+^ were expressed as (*F*−*F*_min_)/(*F*_max_−*F*) according to the equation [Ca^2+^]=*K*_d_ × (*F*−*F*_min_)/(*F*_max_−*F*). *F*_max_ was obtained when cells were treated with the cell-permeable Ca^2+^ ionophore ionomycin which both inserts into the plasma membrane and passes into the cell, inserting into mitochondrial membranes^58^, leading to saturation of the indicator when in regular medium (2 mM Ca^2+^). *F*_min_ was obtained under the same conditions except in zero Ca^2+^ medium. The linear relationship between [Ca^2+^] and (*F*−*F*_min_)/(*F*_max_−*F*) was confirmed by calibrating the indicator as expressed in neurons, exposing them to ionomycin in the presence of sequentially different solutions of precise [Ca^2+^], obtained by mixing K_2_EGTA and CaEGTA solutions (Calcium Calibration Buffer Kit, Invitrogen) at different ratios (data not shown). (c) 4mtD3cpv imaging was performed as described^25^. Fluorescence images were acquired at 2 Hz using a cooled CCD camera (iXon, Andor) through a × 20 water-immersion objective (XLMPlanFluor, Olympus) on an upright microscope (BX51W1, Olympus). Fluorescence excitation (CFP 430±12; YFP 500±10) was provided by a xenon arc lamp in combination with an excitation filter wheel (cell^R, Olympus). CFP (470±12) and YFP (535±15) emission wavelengths were separated and filtered using a DualView beam splitter (MAG Biosystems). Data were collected using proprietary software (cell^R, Olympus), and analyzed using ImageJ and IgorPro (WaveMetrics). When appropriate, only cells co-expressing both a Ca^2+^ indicator and an mCherry or tDimer tag were chosen for analysis. Ca^2+^ concentration changes were quantified using the crosstalk- and bleaching-corrected FRET ratio^25^. Mitochondrial Ca^2+^ responses to NMDA were expressed as (%*R*_fret_/(Δ*R*_max_−%*R*_fret_))^1/n^ according to the equation [Ca^2+^]=*K*′_d_ × (%*R*_fret_/(Δ*R*_max_−%*R*_fret_))^1/n^^25^, where %R_fret_ represents the percent of the maximum response obtained in SGG medium containing 10 μM ionomycin and 10 mM Ca^2+^. The values for DR_max_ and n (105.3% and 0.74, respectively) were derived from published calibrations^25^. The linear relationship between [Ca^2+^] and (%*R*_fret_/(D*R*_max_−%*R*_fret_))^1/n^ was confirmed in neurons exposed to ionomycin in the presence of sequentially different solutions of precise [Ca^2+^], obtained by mixing K_2_EGTA and CaEGTA solutions (Calcium Calibration Buffer Kit, Invitrogen) at different ratios (data not shown). Control values for mitochondrial [Ca^2+^] obtained using 4mtD3cpv were subject to some culture-to-culture variation, which is likely to be due to the fact that it is operating closer to it's maximum than GCaMP2-mt is. As a result of this, we have normalized all 4mtD3cpv [Ca^2+^] data to the average NMDA-induced [Ca^2+^] obtained in control cells on that day of recording. This allows us to directly assess the effect of Mcu expression, Mcu knockdown, or BiC pre-treatment on NMDA-induced mitochondrial [Ca^2+^], expressed as a percentage of the NMDA-induced mitochondrial [Ca^2+^] level obtained in that exact day of recording.

### Mitochondrial membrane potential imaging

Mitochondrial membrane potential was analyzed as described^10^,^28^ using Rh123 (Molecular Probes). Briefly, neurons were loaded with Rh123 (10 μg ml^−1^ or 26 μM) in SGG medium for 10 min followed by extensive washing with SGG. Rh123 partitions into polarized mitochondria where it self-quenches at the concentration used. When mitochondria depolarize, Rh123 leaks out of the mitochondria into the cytoplasm where it dequenches and fluoresces strongly. Maximum Rh123 signal (ex 480±20; em 527±15) was obtained by completely eliminating the mitochondrial potential by exposing the neurons to the mitochondrial uncoupler FCCP (5 μM; Sigma). For analyzing the effect of transfecting Mcu expression vectors or Mcu-directed siRNA, transfected cells were identified by co-transfecting mCherry expression plasmid. The NMDA-induced increase in Rh123 signal of each transfected cell (as a % of the FCCP-induced level for that cell) was compared with that observed in surrounding untransfected cells in a 100-μm radius and subsequently expressed as a percentage of that observed in those untransfected cells. The NMDA-induced increase in Rh123 signal in Mcu-1 shRNA-infected hippocampal neurons was compared with that observed in Scr shRNA-infected cultures analyzed in parallel.

### Studying transfected neurons after excitotoxic insult

Excitotoxicity experiments performed on Lipofectamine-transfected neurons were performed as described^45^,^59^ (for full details see Supplementary Methods). The induction and analysis of NMDA-induced neuronal cell death of rAAV-infected neurons were performed as described^60^, with slight changes. For full details, see Supplementary Methods. Also see Supplementary Methods for details of methodology used to calculate whether Mcu overexpression specifically rendered neurons more vulnerable to NMDA exposure, taking into account the increased basal death observed.

### Quantitative reverse transcriptase PCR

To determine *Mcu* silencing by the pre-made *Mcu*-directed siRNA, and study activity-dependent *Mcu* regulation in cortical neurons, QRT–PCR was performed using an Mx3000P QPCR System as described^45^. For further details including primer sequences, see Supplementary Methods. To determine Mcu silencing by *Mcu*-directed rAAV–shRNA, and study activity-dependent *Mcu* regulation in hippocampal neurons, QRT–PCR was performed using real-time TaqMan technology with a sequence detection system model 7300 Real Time PCR System (Applied Biosystems, Foster City, CA, USA). For further details including primer sequences see Supplementary Methods.

### Statistical analysis

Statistical testing involved a two-tailed paired or unpaired Student’s *t-*test, as appropriate. For studies employing multiple testing, we used a one-way ANOVA followed by Bonferroni *post hoc* test. All data were presented as mean±s.e.m.

## Author contributions

G.E.H and H.B. conceived the project and designed the research. G.E.H., H.B., P.A.S. and D.J.A.W. supervised the research. J.Q., Y-W.T., A.M.H., M.M., N.K. and G.E.H. performed the experiments. G.E.H. and H.B. wrote the manuscript.

## Additional information

**How to cite this article:** Qiu, J. *et al*. Mitochondrial calcium uniporter Mcu controls excitotoxicity and is transcriptionally repressed by neuroprotective nuclear calcium signals. *Nat. Commun.* 4:2034 doi: 10.1038/ncomms3034 (2013).

## Supplementary Material

Supplementary Information Supplementary Figures S1-S5 and Supplementary Methods
