# Supplementary material for: Mitochondrial calcium uniporter Mcu controls excitotoxicity and is transcriptionally repressed by neuroprotective nuclear calcium signals
Source: Nat Commun. 2013 Jun 18;4:2034. doi: 10.1038/ncomms3034 (PMC3709514; doi:10.1038/ncomms3034)
Supplement: Supplementary Information — Supplementary Figures S1-S5 and Supplementary Methods [file ncomms3034-s1.pdf]

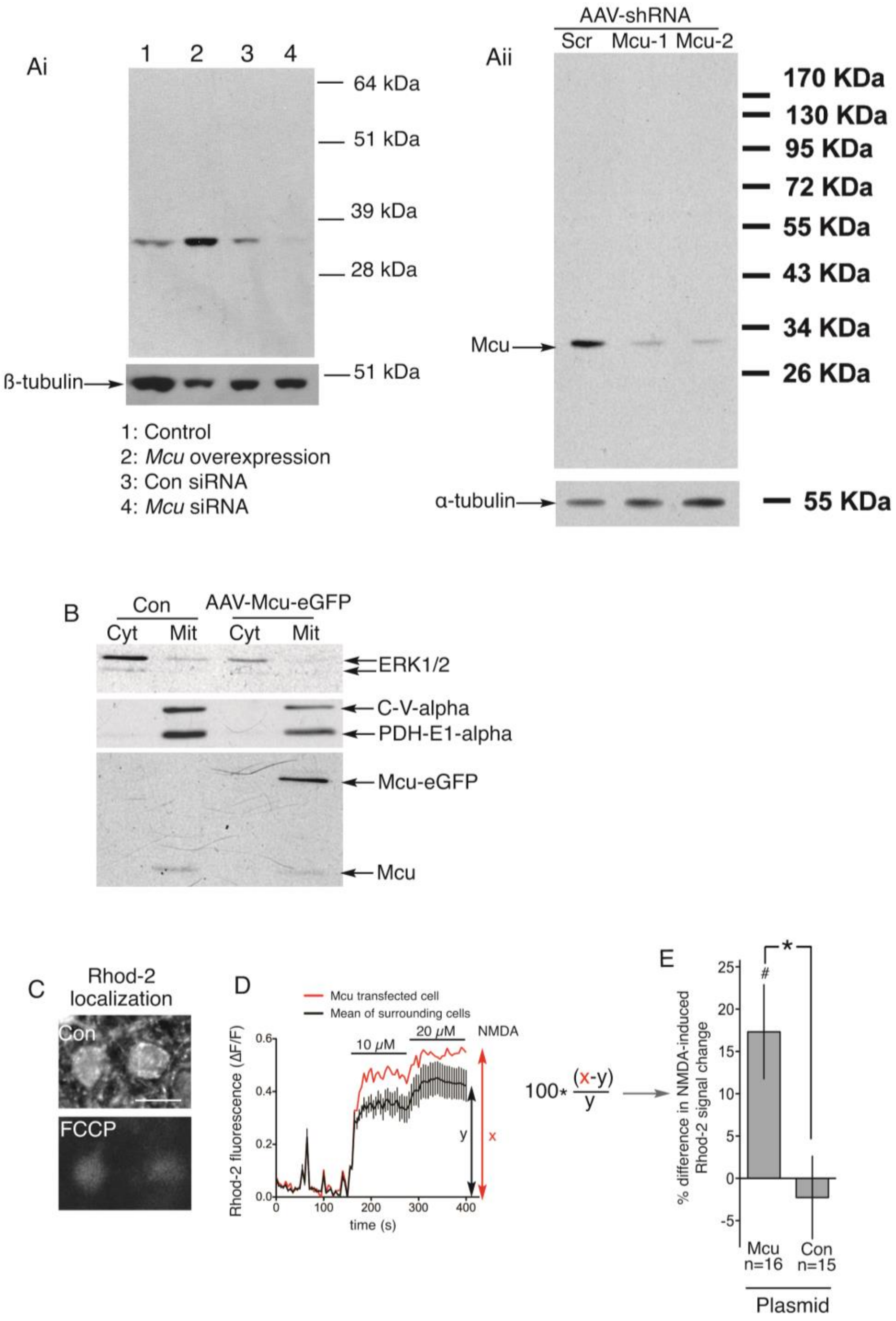

Supplementary Figure S1. Exogenously and endogenously expressed Mcu are mitochondrially localized, and Mcu overexpression increases mitochondrial Ca^{2+} levels following NMDA stimulation. A) Illustration of the specificity of the anti-Mcu antibody. i) Cortical neurons were nucleofected with either a Mcu expression vector or the indicated siRNAs. ii) Hippocampal neurons were infected with AAV containing vectors encoding the indicated shRNAs. **B)** Over-expressed and endogenous Mcu localizes to mitochondria. Neurons were infected where indicated with AAV-Mcu-eGFP and mitochondrial and cytoplasmic fractions prepared, followed by immunoblotting using antibodies against Mcu, mitochondrial markers PDH-E1-alpha and C-V-alpha, plus cytoplasmic proteins ERK1/2. **C)** Image illustrating mitochondrial localization of Rhod-2 ± the uncoupler FCCP. Scale bar = 50

µm. **D,E)** Mcu over-expression boosts mitochondrial Ca^{2+} uptake assayed using Rhod-2. **D)** Example trace from a single experiment where Rhod-2 fluorescence changes (ΔF/F) following NMDA application are studied in a cell over-expressing Mcu (plus a eGFP marker) and surrounding untransfected cells. For each experiment, the difference between transfected and untransfected cells was calculated as shown, and combined with other repeats to give the quantitation shown in **(F)**. # indicates a significant difference between Mcu-expressing cells and surrounding untransfected cells (paired 2-tailed *t*-test, $p<0.05$). *indicates a difference between data obtained with Mcu-expressing cells and that with control-transfected cells ($p<0.05$, unpaired 2-tailed *t*-test n=16 Mcu, n=15 con). Mean ± s.e.m. shown.

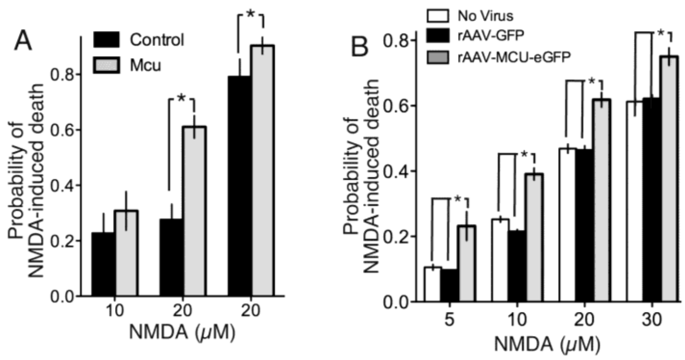

Supplementary Figure S2. Probability of NMDA-induced neuronal death, taking into account changes in basal cell death. See methods for details. (A) relates to Mcu-transfected cortical neurons (Fig. 3a). (B) related to rAAV-Mcu infected hippocampal neurons (see Fig. 3c). Mean ± s.e.m. shown.

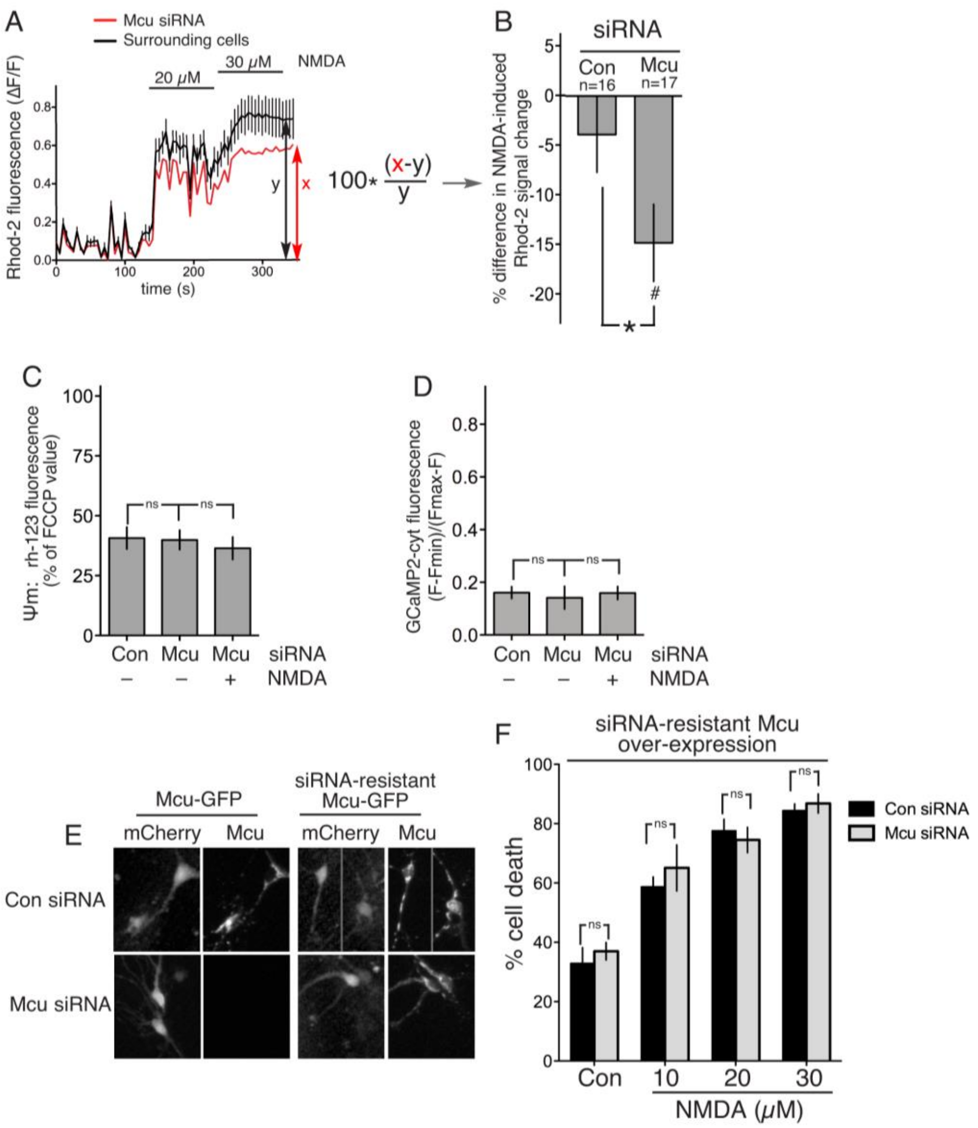

Supplementary Figure S3. Mcu silencing inhibits mitochondrial Ca^{2+} uptake and prevents delayed mitochondrial depolarization and Ca^{2+} deregulation. A,B) Mcu knock-down inhibits mitochondrial Ca^{2+} uptake assayed using Rhod-2. Methodology exactly as for Fig. 1e,f. # indicates a significant difference between Mcu-siRNA transfected cells and surrounding untransfected cells ($p<0.05$, paired 2-tailed *t*-test). * indicates a significant difference between data obtained with Mcu-siRNA transfected cells and that obtained from control-transfected cells ($p<0.05$,unpaired 2-tailed *t*-test n=17 Mcu siRNA, n=16 con siRNA). Mean ± s.e.m. shown. **C)** Mitochondrial membrane potential is preserved in neurons protected from excitotoxicity by Mcu knockdown. Neurons were transfected with the indicated siRNA and treated ± NMDA as indicated, following the same protocol as Fig. 4i. 24 h after NMDA treatment, mitochondrial membrane potential in transfected neurons was measured by loading them with rhodamine-123 and measuring fluorescence as a % of that obtained upon mitochondrial depolarization with FCCP (15-22 cells analysed per condition within n=3 independent experiments). **D)** Normal resting Ca^{2+} levels are observed in neurons protected from excitotoxicity by Mcu knockdown. Neurons were transfected with the indicated siRNA, plus an expression vector for cyt-GCaMP

and treated ± NMDA as indicated, following the same protocol as Fig. 4i. 24 h after NMDA treatment, cytoplasmic Ca2+ levels were calculated from the cyt-GCaMP signal followed by calculation of F_{max} and F_{min} as per the methods (10-24 cells analysed per condition). **E)** An siRNA-resistant form of Mcu-GFP was synthesized containing four (conservative) mutations within the siRNA target region. Resistance to the siRNA was confirmed by transfecting neurons with an mCherry vector plus either wild-type or siRNA-resistant Mcu-GFP in the presence of either control or the Mcu-directed siRNA. As illustrated, Mcu siRNA prevents expression of regular Mcu-GFP but not the siRNA-resistant form. **F)** Over-expression of siRNA-resistant Mcu reverses the protective effect of Mcu siRNA. Performed as in Fig. 4i except that neurons were additionally transfected with siRNA-resistant Mcu-GFP (n=3).

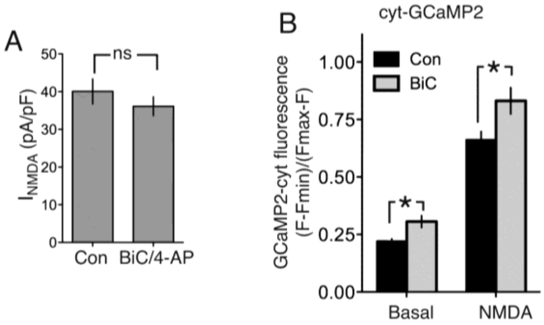

Supplementary Figure S4. NMDAR whole-cell currents and cytoplasmic Ca^{2+} are not reduced by BiC/4-AP pre-treatment. A). Whole-cell NMDAR currents recorded in cortical neurons in the presence or absence of overnight BiC/4-AP pre-treatment (n=10 (Con), n=12 (BiC/4-AP). Mean ± s.e.m. shown. **B)** Neurons were treated and studied exactly as in Fig. 6c except that they were transfected with a non-tagged, cytoplasmic form of GCaMP2. *p<0.05 (unpaired 2-tailed *t*-test, Con: 54 cells, n=7; BiC/4-AP: 78 cells, n=7). Mean ± s.e.m. shown.

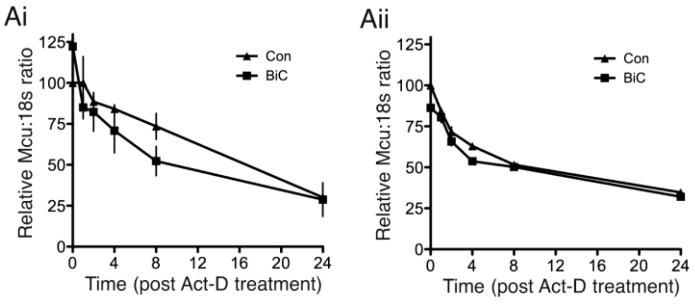

Supplementary Figure S5. The decay kinetics of Mcu mRNA are not affected by BiC/4-AP pre-treatment. Cortical (i) and hippocampal (ii) neurons were treated ± BiC/4-AP for 30 min, at which point transcription was blocked by treatment with Actinomycin D (10 µg/ml) for varying times as indicated. RNA was then harvested and expression of Mcu mRNA was measured relative to a relatively stable RNA (18S rRNA). This number was then normalized to the t=0 control value. Mean ± sem of n=3 is shown.

Supplementary Methods

Subcellular fractionation.

Fractionation of mitochondrial and cytoplasmic fractions was performed using the Cell Fractionation kit (Abcam, ab109719) according to the manufacturer's instructions with slight modifications. Briefly, mouse hippocampal neurons (0.5 x 10^6) were washed with PBS and harvested into 350 µl ice-cold PBS containing 1x protease inhibitor cocktail (Complete, Roche). The cells were washed with 1 ml Buffer A and resuspended in 350 µl Buffer A. After Detergent I treatment for 20 min at RT, cells were centrifuged to collect cytosolic proteins. The pellet was re-suspended in Buffer A and treated with Detergent II for 10 min at RT. The suspension was centrifuged and supernatant was collected as mitochondria fraction.

Transfection and nucleofection

Neurons were transfected in trophic transfection medium [55] with plasmids (2µg/ml total) and/or siRNA (100nM) using Lipofectamine 2000. The sequence targeted by Mcu-directed siRNA is cgaccuagagaaauacaau; control siRNA used was siGENOME Non-Targeting siRNA 2 (Dharmacon). Experiments were performed 48h post-transfection. Nucleofection was performed using the Amaxa rat Neuron Nucleofector Kit (Lonza). Briefly, for every sample, mouse cortical neuron pellets containing 4-5 x 10^6 cells were mixed with 100µl nucleofector solution and either 3µg plasmid DNA or 300µmol siRNA. Nucleofection was carried out using a Nucleofector II system. Following nucleofection, 50µl pre-equilibrated Opti-MEM was added to each sample and the cells gently plated down in 24-well dish. Nucleofection efficiency was determined by eGFP expression 48 h after transfection and was routinely 50%-60%.

Plasmids and virus generation

The vector containing the mouse CaMKIIα promoter used to construct and package recombinant adeno-associated virus (rAAV) has been described previously [56]. A full-length mouse Mcu (NM_001033259) without a stop codon was amplified from the commercial plasmid (OriGene, MC212635) by PCR using primers: Fwd: 5’-ttcgtgcgcggatcc

accatggcggccgccgcaggtagat-3'; Rev: 5'-ccctgccagggctagcttccttttctccgatctgtc-3'. The PCR fragment was cloned into rAAV vectors with an eGFP tag or tDimer tag via BamHI and NheI sites. Similarly, full-length 4mtD3cpv was amplified from an mt-cameleon-pcDNA3 plasmid by PCR and inserted into the rAAV vector using AgeI and EcorI sites: Fwd: 5'-gccgacgaccggtcgactcactatagggagacccaagcttat-3'; Rev: 5'-cggccgccagtgtgatggat-3'. The same vector was used to express eGFP. We used a rAAV vector containing the U6 promoter driving shRNA expression and that also contained a CaMKII promoter driving mCherry expression (to identify infected neurons). For silencing Mcu, we used two hairpins: Mcu-1 (from the TRC, TRCN0000251263: tagggaataaagggatcttaa); Mcu-2 (gggcttagcgagtcttgtc). For silencing Npas4, two hairpins targeted to the 3' UTR of the mouse Npas4 mRNA were used: Npas4-sh1 (from the TRC, TRCN0000424907, atggatttcaagcggagaatg), Npas4-sh2 (tctgtgacttaacgtcttcaa) [35]. The non-targeting shRNA (scrambled) is the following: gtgccaagacgggtagtca. All rAAV vectors were generated by standard molecular biology techniques and verified by sequencing. Viral particles were produced and purified as described previously [56]. Neurons were infected with 10^{11} rAAV particles/μL at DIV 4. Infection efficiencies were determined at DIV 10 by analyzing the fluorescence of eGFP or mCherry; they ranged from 80 to 90% of the viable neurons. rAAV expression vectors for Npas4, CaMBP4 and mCherry have been described previously [35, 56]. Mcu-GFP was a gift from Vamsi Mootha [18], pCAGGS-GCaMP2 was a gift from Karel Svoboda [57]; GCaMP2-mt was a gift from Xianhua Wang [24], Mt-cameleon-pcDNA3 (containing 4mtD3cpv cDNA) was a gift from Roger Tsien [25].

Studying transfected neurons after excitotoxic insult:

Excitotoxicity experiments performed on Lipofectamine-transfected neurons were performed as described [45, 59]. Neurons were transfected with the plasmid or siRNA of interest (or control) plus eGFP marker. To apply an excitotoxic insult, neurons were first placed overnight into a minimal defined medium [33, 45]. Pictures were then taken using a Leica AF6000LX system (DFC350 FX camera) prior to NMDA treatment for 1 h, after which 10 μM MK-801 was added. Using cell-finder software, images of the same neurons were taken 24 h post-insult. Cell death was determined by counting the number of surviving GFP-expressing cells post-insult. Death was

indentified by the absence of healthy GFP-expressing neurons: in >90% of cases fluorescent cell debris and fragmented nuclei were observed. For each condition, the fate of 300-600 neurons was monitored over n=4-6 experiments done on independent cultures. The induction and analysis of NMDA-induced neuronal cell death of rAAV-infected neurons was done as described [60] with slight changes. Briefly, neurons were treated with NMDA with different concentrations for 10 min at 37°C, washed three times with transfection medium [55] and incubated at 37°C for 20 h. The percentage of dead cells was determined by analyzing Hoechst 33258 stained nuclei.

In the case of Mcu-over-expressing neurons, we found that this increased basal levels of death (in the absence of NMDA exposure). In addition to showing the raw cell death data (Fig. 3), we also calculated whether Mcu overexpression specifically rendered neurons more vulnerable to NMDA exposure, taking into account this increased basal death (Supplementary Fig. S2). The chances of a neuron surviving an NMDA insult in the presence or absence of Mcu expression can be expressed according to the equation: $P_{SURVIVAL} = (1-P_{BASAL}) \times (1-P_{NMDA})$, where P_{BASAL} is the probability of a neuron dying in the absence of any NMDA, and P_{NMDA} is the probability of a cell dying due to the NMDA insult. A value for P_{NMDA} can be calculated because we know the other variables in the equation (we know $P_{SURVIVAL}$ from the cell death data and P_{BASAL} from the cell death % in the absence of NMDA).

Quantitative reverse transcriptase PCR

To determine *Mcu* silencing by the pre-made *Mcu*-directed siRNA QRT-PCR was performed using an Mx3000P QPCR System. Following nucleofection of control or Mcu-directed siRNA, RNA was isolated using Qiagen RNeasy isolation reagents, cDNA prepared and qPCR performed as described [45]. Technical replicates as well as no template and no RT negative controls were included and at least 3 biological replicates were studied in each case. The sequences of the primers used are as follows Mcu: Fwd: 5'-cgccaggaatatgtttatcca-3'; Rev: 5'-cttgtaatgggtctctcagtctctt-3'. GAPDH: Fwd: 5'-cttcaccaccatggagaaggc-3'; Rev: 5'-ggcatggactgtggtcatgag-3'. The data were analysed using the MxPro QPCR analysis software (Stratagene). Expression of Mcu was normalized to Gapdh. To determine Mcu silencing by

Mcu-directed rAAV-shRNA, QRT-PCR was performed using real-time TaqMan technology with a sequence detection system model 7300 Real Time PCR System (Applied Biosystems, Foster City, CA, USA). Total RNA was extracted using RNeasy kit (Qiagen GmbH, Germany) with additional on-column DNase I digestion during RNA purification. cDNA was generated from 1 μg of total RNA using High Capacity cDNA Reverse Transcription kit (Applied Biosystems). QRT-PCR was carried out using TaqMan Universal PCR Master Mix (Applied Biosystems). The following TaqMan gene expression assays were used in this study: *Mcu* (Mm01168773_m1), *gusb* (Mm00446953_m1), *Npas4* (Mm00463644_m1), *18 s rRNA* (4319413E). The expression levels of the target mRNA were normalized to the relative ratio of the expression of Gusb or *18 s rRNA* as endogenous control.
